# Surgical procedure of canaliculoplasty in the treatment of primary canaliculitis associated with canalicular dilatation

**DOI:** 10.1186/s12886-020-01503-z

**Published:** 2020-06-20

**Authors:** Yun Su, Leilei Zhang, Lunhao Li, Xianqun Fan, Caiwen Xiao

**Affiliations:** 1 Department of Ophthalmology, Shanghai Ninth People’s Hospital, Shanghai Jiao Tong University School of Medicine, No. 639 Zhizaoju Road, Shanghai, 200011 China; 2 Shanghai Key Laboratory of Orbital Diseases and Ocular Oncology, Shanghai, China

**Keywords:** Primary canaliculitis, Canaliculoplasty, Epiphora, Dacryoendoscopy

## Abstract

**Background:**

Primary canaliculitis is a chronic infection of the proximal lacrimal pathway. We aimed to evaluate surgical outcomes of a canaliculoplasty procedure for primary canaliculitis associated with canalicular dilatation.

**Methods:**

This study enrolled 42 primary canaliculitis patients with canalicular dilatation who underwent canaliculoplasty. All patients were treated with canaliculotomy, curettage of canalicular contents and canaliculoplasty with stent placement. Patients’ demographics, clinical features, and follow-up outcomes were evaluated.

**Results:**

There were 12 males and 30 females with a mean age of 66.1 ± 13.9 years. The mean duration time from the first onset of signs/symptoms to diagnosis was 30.6 ± 39.5 months. Epiphora (90.5%) and mucopurulent discharge from punctum (85.7%) were the most common signs. Thirty-three out of 42 patients (78.6%) achieved complete remission with a mean follow-up time of 25.3 ± 12.9 months. There were 3 patients found to have canalicular stenosis due to obstruction after surgery.

**Conclusion:**

Canalicular dilatation is a severe condition of primary canaliculitis, probably due to a combined result of long standing disease and the presence of concretions. The surgical procedure of canaliculoplasty can be a highly effective treatment for primary canaliculitis associated with canalicular dilatation.

## Background

Primary canaliculitis is a chronic infection of the proximal lacrimal pathway [1]. Patients with canaliculitis typically present with epiphora, mucopurulent discharge and a pouting punctum. These patients are often misdiagnosed with conjunctivitis, chalazion or dacryocystitis [2], which may lead to delayed or even wrong treatment [3].

Surgical removal of all possible concretions is considered essential for permanent cure and has been proven to have clear benefits over conservative management [4–6]. Surgical managements, such as punctoplasty, pure canaliculotomy, and canalicular curettage, are common methods for treating primary canaliculitis. However, in patients with canalicular dilatation due to a long-term stasis of concretions, these methods have their limitations, because of the failure in the reconstruction of anatomical structure of canaliculus, which would result in recurrence of primary canaliculitis [6–8].

In this study, we reported long-term outcomes and efficacy of our surgical technique, which included a procedure of canaliculotomy, curettage of canalicular contents and canaliculoplasty with stent placement for the treatment of primary canaliculitis associated with canalicular dilatation.

## Methods

The medical records of patients diagnosed as primary canaliculitis with canalicular dilatation and achieved surgical treatment in Shanghai Ninth People’s Hospital from January 2011 to June 2018 were reviewed. This study followed the tenets of the Declaration of Helsinki and was approved by the Ethics Committee of Shanghai Ninth People’s Hospital.

Canaliculitis was diagnosed based on patients’ clinical histories and findings. Dacryoendoscopy was performed in all patients and those with expansion of the canalicular lumen were diagnosed as canalicular dilatation. The following data were collected, including clinical presentations, intraoperative findings, microbiological and histological analyses, follow-up information and clinical outcomes. Patients without canalicular dilatation, with secondary canaliculitis (e.g. punctual plug-related canaliculitis) or with previous lacrimal surgeries were excluded from this study.

The surgical procedures are well known in the literature [9–11]. In brief, following local anesthesia, a canaliculotomy incision was made through conjunctival approach parallel to the dilated horizontal canaliculus, and curettage was performed to evacuate concretions inside the canaliculus. The excess canalicular tissue due to dilatation was excised. A Crawford bicanalicular silicone stent (Shandong Bausch & Lomb Freda, Shandong Province, China) was placed and tied in the inferior meatus to prevent stent prolapse or stent loss. Then canaliculoplasty was performed using an 8–0 polyglactin suture. After that, a 6–0 polyglactin suture was tied around the ampullary part to prevent punctal slitting. The concretions and excess canalicular tissues were submitted for microbiologic culture and histological diagnosis. After surgery, patients were prescribed with topical levofloxacin drops 4 times per day for 3 weeks. The type and dosage of antibiotics might be adjusted according to the results from microbiologic culture and drug sensitivity test [9, 12, 13]. The silicone stent was removed 6 to 8 months later.

## Results

Forty-two primary canaliculitis patients were found with canalicular dilatation and underwent canaliculotomy followed by canaliculoplasty. There were 30 women and 12 men with a mean age of 66.1 ± 13.9 years old, ranging from 28 to 87 years. There were 26 patients with only right eye involvement, 13 with only left eye involvement and 3 with both eyes. Their clinical characteristics were summarized in Table 1. The mean duration time from the first onset of symptoms/signs to diagnosis was 30.6 ± 39.5 months, ranging from 3 months to 20 years. Epiphora and mucopurulent discharge from punctum were the most common clinical presentations noted in 38 (90.5%) and 36 (85.7%) patients, respectively (Table 2). Lacrimal drainage irrigation was patent before surgery in all patients. No patients had mucous reflux when compressed lacrimal sac.

**Table 1:** Clinical characteristics of primary canaliculitis patients associated with canalicular dilatation

	No. (%)
Gender	
Male	12 (28.6)
Female	30 (71.4)
Age (years, range)	66.1 ± 13.9 (28 to 87)
Mean time to diagnosis (months, range)	30.6 ± 39.5 (3 months to 20 years)
Location	
Upper canaliculus only	6 (14.3)
Lower canaliculus only	32 (76.2)
Both	4 (9.5)
Laterality	
Right	26 (61.9)
Left	13 (31.0)
Both	3 (7.1)
Mean follow-up (months, range)	25.3 ± 12.9 (1 year to 6 years)

**Table 2:** Clinical presentations of primary canalicutlitis patients associated with canalicular dilatation

Clinical Presentations	No. (%)
Symptoms	
Epiphora	38 (90.5)
Mucopurulent discharge	31 (73.8)
Conjunctival injection	10 (23.8)
Pain	12 (28.6)
Eyelid redness and swelling	15 (35.7)
Signs	
Discharge from punctum	36 (85.7)
Pouting punctum	14 (33.3)
Palpable thickened canaliculus	16 (38.1)
Punctal erythema and swelling	25 (59.5)

All patients underwent surgical treatment under local anesthesia. The contents in the canaliculus was removed after a horizontal canaliculotomy. The concretions were taken out during surgery and submitted for aerobic, anaerobic and fungal cultures according to standard microbial procedures, and also for the histological examination. Microbiologic cultures demonstrated positive results in 10 patients (23.8%), including *Streptococcus species* in 4 patients, *Actinomyces species* in 3 patients and *Staphylococcus species* in 3 patients. For the rest of the patients, no pathogenic organism was identified from the concretions. Histological diagnosis of concretions by Gomori methenamine silver stain revealed *Actinomyces species* in 2 patients. In the rest of the patients, only inflamed granulation tissues were found. After canaliculotomy and thorough curettage of the concretions, we found polypoid changes of the canalicular mucosae in 34 (81.0%) patients by dacryoendoscopy, mainly involving the distal part of the horizontal canaliculus. Histology of the excised tissues revealed polypoid epithelial hyperplasia and diffuse infiltration of chronic inflammatory cells. Their microbiologic cultures were negative.

The follow-up time after surgery ranged from 1 year to 6 years with a mean time of 25.3 ± 12.9 months. Three months after surgery, there were 33 (78.6%) out of 42 patients achieving complete remission of their clinical symptoms and signs. The remaining 9 patients still presented with different degrees of epiphora without mucopurulent discharge. Their mean duration time from the first onset of symptoms to surgery was 66.2 months. Lacrimal drainage irrigation was patent in 6 of these patients and the other 3 (mean age 52.0 years) were found to have complete canalicular obstruction due to scar formation by dacryoendoscopy. No further treatment was given to these patients. During follow-up visits, no patient developed recurrence of canaliculitis after surgery.

## Discussion

Primary canaliculitis is a rare disease, accounting for 0.81 to 2% of all patients with lacrimal disease [1, 14]. Although the clinical presentations have been documented in many studies, there is still a high rate of delayed or wrong diagnosis due to a lack of typical clinical features [15–17]. In this study, we paid special attention to the patients who had already been delayed for many years, and all of them were diagnosed with severe primary canaliculitis with associated canalicular dilatation.

**Fig. 1:**
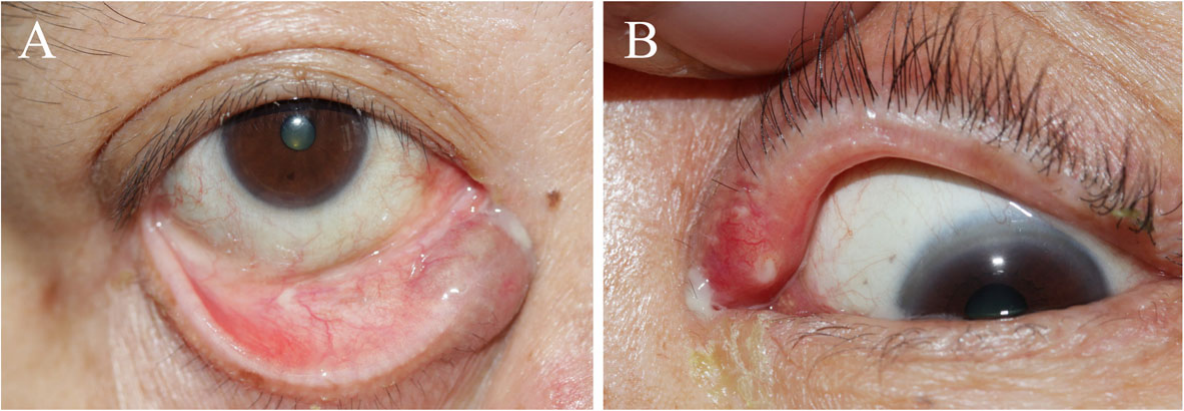
Clinical signs of primary canaliculitis with canalicular dilatation. **a** Lower punctal edema, mucopurulent discharge and palpable thickened canaliculus. **b** Upper punctal erythema and edema, mucopurulent discharge from punctum and conjunctival injection

Our series of patients were diagnosed with canaliculitis based on their clinical histories and findings (Fig. 1). There was no apparent difference between canaliculitis patients with and without canalicular dilatation in their clinical presentations. Mucopurulent discharge is more common in patients with canalicular dilatation, but it is not a determinant factor for diagnosis. Palpable thickened canaliculus could be a sign of canalicular dilatation. Canalicular dilatation may be a consequence of a long-term stasis of concretions during the course of canaliculitis. Yuksel et al. [18] reported two patients with severe canalicular dilatation with a duration time of 7 years and 3 years, respectively. The mean duration time to diagnosis was 30.6 ± 39.5 months in our study, which is longer than others. Longer duration time may probably add to the severity of the clinical presentation.

*Actinomyces* was considered the most common pathogenic bacteria in canaliculitis [12, 19, 20]. Canalicular concretions and the presence of sulfur granules in histology were considered in association with *Actinomyces* [10, 12, 21], but not necessarily indicated to *Actinomyces* infection [22]*.* In this study, only three patients were positive for *Actinomyces species*. Such low prevalence is in concordance with the findings from other Asian countries, where other pathogens are more commonly seen than *Actinomyces*. For example, *Streptococcus species* was more common in canaliculitis samples in some studies [5, 7, 23]. In addition, several studies have also reported some rare organisms that can cause canaliculitis [24, 25]. The positive rate of bacterial cultures in this study (23.8%) was lower than other reports [12, 23]. Given the long duration time before diagnosis, our hypothesis suggested that in some cases, the long-lasting inflammation was more likely contributed by the concretions and thick mucopurulent discharge compared to the infection of bacteria itself.

**Fig. 2:**
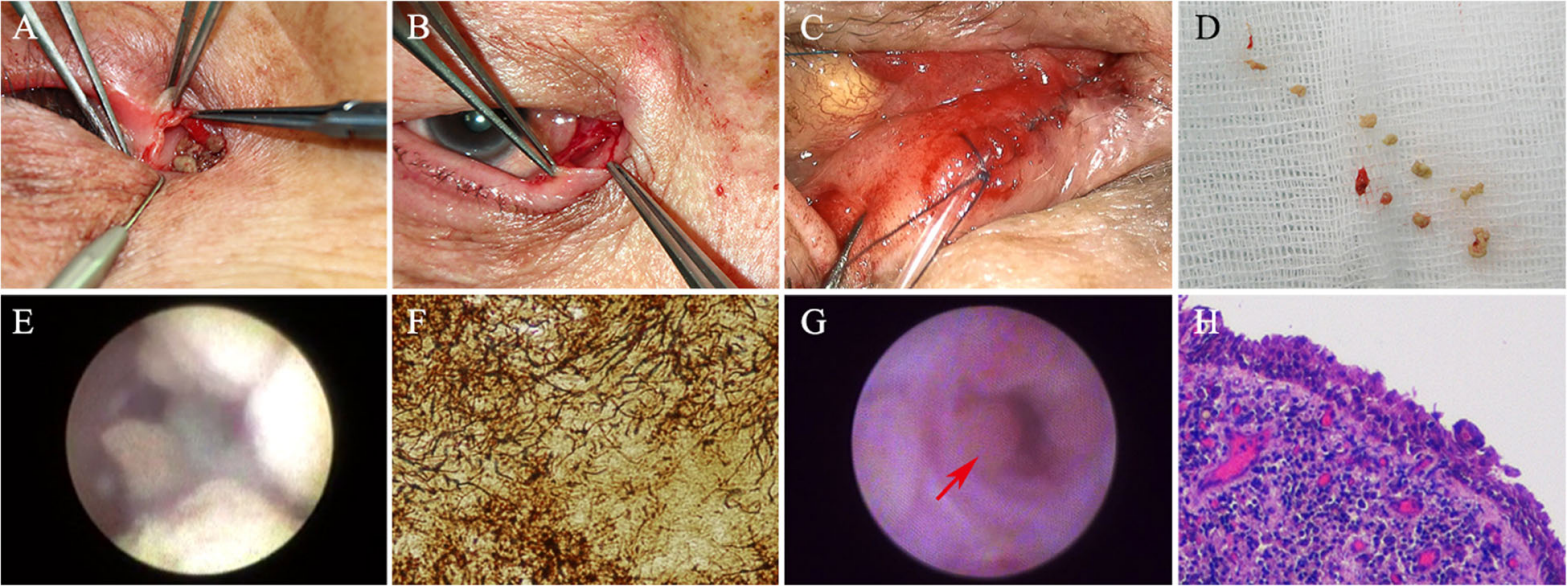
Intraoperative and histological findings of primary canaliculitis with canalicular dilatation. **a** Concretions inside the lumen after canaliculotomy of the lower horizontal canaliculus. **b** Dilatation, erythema and edema of the upper canalicular lumen. **c** Curettage of the concretions. **d** After canaliculoplasty, a 6–0 polyglactin suture was tied around the ampullary part to prevent punctal slitting. **e** Dacryoendoscopic photograph showing expansion of the canalicular lumen, edematous mucosa of the canaliculus with yellowish and fluffy concretions around the canalicular walls. **f** Histological examination of concretions, showing *Actinomyces species* infection. (Gomori methenamine silver stain, × 400) **g**. Dacryoendoscopic photograph showing polypoid change of the canalicular mucosae (arrow) after removal of concretions. **h** Histological examination of canalicular mucosae, showing infiltration of inflammatory cells. (**h**&**e**, ×100)

Dacryoendoscopy allows direct visualization of the lacrimal drainage system and can facilitate the morphological assessment of canaliculitis. It can also help with the diagnosis and localization of the lesions within the canaliculus, as well as evaluation of degrees of canalicular expansion [26]. Ali et al. [27] described their dacryoendoscopic findings and reported two types of concretions with different sizes, borders and locations in a 65-year-old female patient without canalicular dilatation. The large ill-defined concretions were found adjacent to the mucosal wall, while the small well-defined ones were in the central area. The patient was then successfully treated with non-incisional canalicular curettage and topical antibiotics. In our study, dacryoendoscopic examination showed expansion of the canalicular lumen, and edema and hyperemia of the mucosae in the vertical and horizontal canaliculi with yellowish concretions on the canalicular walls, which were similar to the previous report [27] (Fig. 2). After removal of concretions, polypoid change of the canalicular mucosae was found. Histology revealed polypoid epithelial hyperplasia and diffuse inflammation which may serve as evidence to a long-term inflammation of canalicular mucosae after infection. Infection of bacteria will cause inflammation of the canalicular mucosae and formation of concretions. The accumulated concretions, together with the mucopurulent discharge, are responsible for a self-perpetuating cycle of canalicular stasis and infection for years [4], thus leading to a long-lasting inflammation and a gradually enlarged canalicular lumen.

The expansion of canalicular lumen mostly occurs in the horizontal canaliculus, starting from the ampulla, noted by dacryoendoscopy. In the observational anatomic study of the lacrimal punctum and canaliculi, Kakizaki et al. [28] found that the horizontal canaliculus was encircled by the Horner muscle, which was relatively weaker than the vertical canaliculus, part of the tarsal plate. So the horizontal canaliculus is more dilatable and accommodative of concretions. Therefore, the purpose of clinical treatment is to eliminate the concretions from the ampulla to the affected horizontal canaliculus.

Different surgical managements, such as canaliculotomy, punctoplasty, and canalicular curettage, have been proven to be effective in primary canaliculitis to varying degrees [4–6, 29]. The aim of all surgical managements is to remove concretions and to eliminate stasis and subsequent bacterial growth. Canaliculotomy allows better access to the canaliculus and more thorough curettage of canalicular contents. Canalicular curettage after canaliculotomy carries a high remission rate and is one of the options for primary canaliculitis [7, 9, 10, 12, 30]. However, in patients with canalicular dilatation, canaliculotomy alone may not be sufficient because it fails to reconstruct the anatomical structure of canaliculus. The expanded lumens and disordered tear flow may lead to recurrence of symptoms. Hence, canaliculoplasty is required.

Canaliculoplasty includes procedures of removing the excess canalicular tissues and placing a stent, which provides an ideal technique to reconstruct the canaliculus without injury to the punctum. The application of stent is usually employed in the situation where patency of the canaliculus is in question [19]. In our study, we regard the stent placement as a good support to maintain the canalicular structure for better integrity and to prevent possible stenosis after surgery. Jin et al. [11] suggested that the intubation could eliminate the abscess cavity and help to clear away pus by the pressure over the affected canaliculus, supported by a possible mechanism that existence of the stents ensured tear drainage by capillary action, leading to an adequate concentration of topical antibiotics and oxygen within the lacrimal system. After intubation, even though some studies do not advocate wound closure by suturing [6, 11], long-term follow-up did not reveal a higher risk of narrowing or lacrimal pump dysfunction in our cases. This technique has the advantage of eliminating the lesions thoroughly, including the concretions and the affected mucosae, with preservation of the integrity of the punctum.

The choice of canalicular stent is usually at the surgeon’s discretion. Monocanalicular stent, such as mini-Monoka® silicone stent, has been reported for intubation after canaliculotomy [31]. Crawford silicone stent, a kind of bicanalicular stent, has also been applied in some studies in the treatment of primary canaliculitis [11, 12]. We also chose Crawford stents mainly because the expansion of lumen mostly occurred in the horizontal canaliculus, starting from the ampulla. The canaliculotomy was performed sometimes even up to the distal part of the horizontal canaliculus to ensure a thorough elimination of concretions. The residual tissues could be bridged better by the bicanalicular stents. Admittedly, bicanalicular stents have been reported to have many complications, including inadvertent damage to the uninvolved canaliculus, false passage, canalicular or punctal slitting, granuloma formation and the presence of biofilms [32–34]. Biofilms have been reported in both cases using mono® and bicanalicular stents. Further studies are needed in this grey area of canalicular stent selection [35–38]. In severe cases of primary canaliculitis with canalicular dilatation, the application of bicanalicular stents still came with favorable treatment outcomes in our study, while in mild or moderate cases of primary canaliculitis, monocanalicular stents may be theoretically more desirable.

**Fig. 3:**
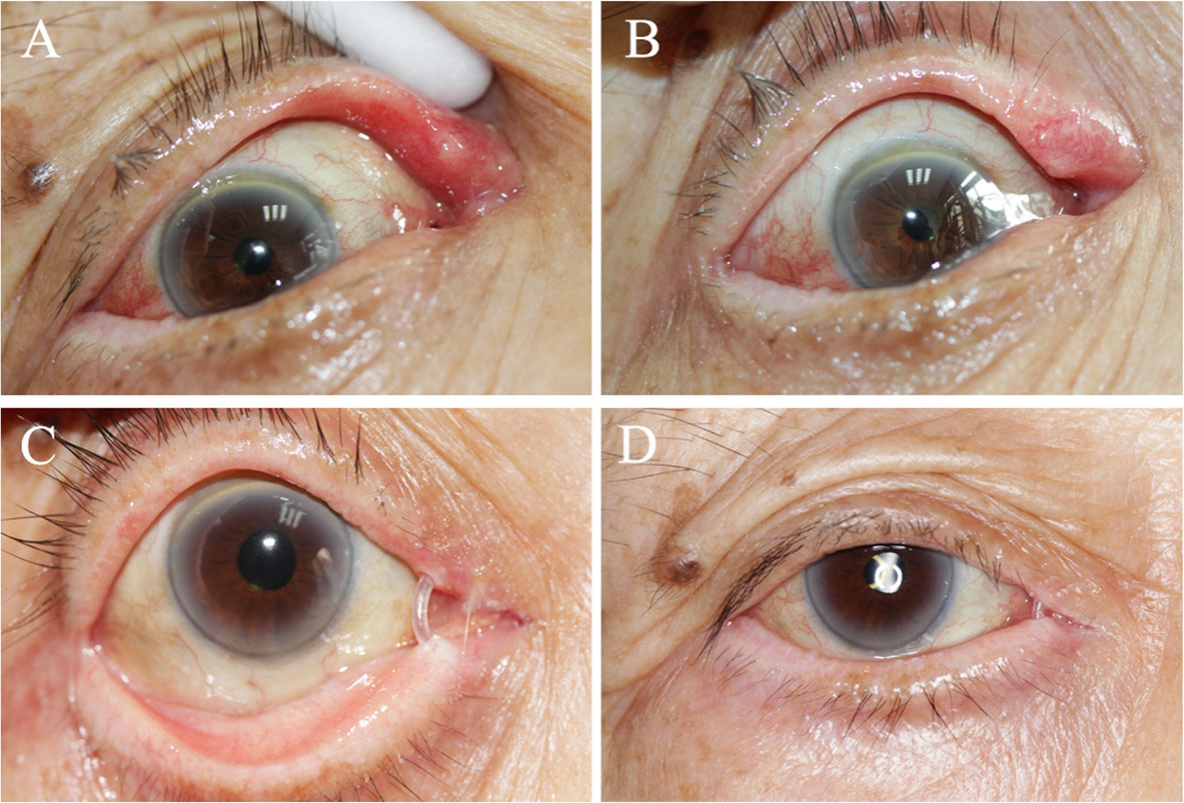
Surgical outcomes of primary canaliculitis with canalicular dilatation. **a**&**b**. Photograph showing the preoperative appearance of the upper punctum of a patient, which is pouting punctum with erythema and edema, and palpable thickened canaliculus. **c**&**d**. Photograph of the same patient 3 months after the surgery. The signs of canaliculitis have resolved completely and the silicone stent is still in place

All patients achieved complete remission of mucopurulent discharge with a mean follow-up of 25.3 ± 12.9 months (Fig. 3). Remission of epiphora was noted in 78.6% (33 out of 42) of patients. The rest 9 patients still presented with epiphora to different degrees. The mean duration time to diagnosis of these 9 patients was 66.2 months, which was much longer than the other cases in our study. The long-term inflammation of the mucosae may lead to injury to the epithelia. In addition, even though canalicular reconstruction has been achieved after canaliculoplasty, lacrimal pump function might have been affected through the long course of disease. Among these 9 patients, there were 3 patients developing symptomatic canalicular obstruction due to scar formation. Their mean age was 52 years old, which was relatively young. The more active tissue repairing and wound healing might play a role in the scar formation. Fortunately, in our long-term follow-up, no patient experienced recurrence after the surgery. Recurrence of canaliculitis has been reported of in several studies. Lin et al. [7] reported a 21% (7 out of 34) recurrence rate after canaliculotomy with a mean follow-up time of 24 months. Xiang et al. [13] also reported their rate of 2.8% (1 out of 36) with 21.7 months follow-up. Our technique appeared to be a decent approach for primary canaliculitis with canalicular dilatation to eliminate the risk of recurrence.

This study is limited by its retrospective nature and the lack of a control group. We haven not compared our technique with others’ due to similar limitation in other studies. We fail to compare the efficacy in patients with and without canaliculoplasty due to the rareness of canalicular dilatation. In addition, the positive rate of microbiologic cultures is low in this study, which prevents us from conducting a comprehensive microbiological analysis.

## Conclusions

In summary, canalicular dilatation is a severe clinical condition of primary canaliculitis. The surgical procedure of canaliculotomy followed by curettage and canaliculoplasty appears to be a highly effective treatment for this disease.

## Data Availability

The datasets analysed during this study are available from the corresponding author on reasonable request.
